# Recent Advances in CXCL12/CXCR4 Antagonists and Nano-Based Drug Delivery Systems for Cancer Therapy

**DOI:** 10.3390/pharmaceutics14081541

**Published:** 2022-07-25

**Authors:** Ruogang Zhao, Jianhao Liu, Zhaohuan Li, Wenhui Zhang, Feng Wang, Bo Zhang

**Affiliations:** School of Pharmacy, Weifang Medical University, Weifang 261053, China; 15264626411@163.com (R.Z.); 17852062086@163.com (J.L.); lzh15098737363@163.com (Z.L.); zwh13869257933@163.com (W.Z.)

**Keywords:** CXCL12/CXCR4 axis, chemokines, cancer therapy, nano-based drug delivery system

## Abstract

Chemokines can induce chemotactic cell migration by interacting with G protein-coupled receptors to play a significant regulatory role in the development of cancer. CXC chemokine-12 (CXCL12) can specifically bind to CXC chemokine receptor 4 (CXCR4) and is closely associated with the progression of cancer via multiple signaling pathways. Over recent years, many CXCR4 antagonists have been tested in clinical trials; however, Plerixafor (AMD3100) is the only drug that has been approved for marketing thus far. In this review, we first summarize the mechanisms that mediate the physiological effects of the CXCL12/CXCR4 axis. Then, we describe the use of CXCL12/CXCR4 antagonists. Finally, we discuss the use of nano-based drug delivery systems that exert action on the CXCL12/CXCR4 biological axis.

## 1. Introduction

Chemokines (CKs) are small cytokines or signaling proteins secreted by cells with a molecular weight of 8–10 kDa with a structure consisting of a short N-terminal region, an extended N loop region, three beta chains, and an alpha helix [1]. The family of chemokines contains approximately 50 species that are classified into four categories based on the number and spacing of nitrogen-terminal cysteines: C, CC, CXCR, and CX3C. Chemokines play an important role in controlling cell proliferation, adhesion, and metastasis during wound healing and embryonic development. Chemokines also regulate many physiological processes, such as the maturation and homing of immune cells, cytoskeletal rearrangements, and immune responses [2]. Furthermore, chemokines play a crucial role in certain stages of tumor development [3,4]. Of these different chemokines, CXCL12 possesses a strong chemotactic ability to cells expressing high levels of CXCR4 [5]. In addition, the interaction between CXCL12 and its receptor CXCR4 has generated widespread interest with regard to tumor progression [6].

## 2. CXCL12/CXCR4 Biological Axis and Its Physiological Functions

The CXCL12/CXCR4 biological axis is a coupled molecular pair, which is formed by the interaction of CXCL12 and its corresponding receptor CXCR4, and is closely related to intercellular messaging and cell migration.

### 2.1. Basic Concepts

Chemokine CXCL12 is a steady-state chemokine that is also known as stromal cell-derived factor-1 (SDF-1). There are multiple isoforms of CXCL12, including α, β, and γ. CXCL12α is rapidly degraded in the blood by protein hydrolysis, while the β isomer is more resistant to blood-dependent degradation and represents a powerful stimulator of neoangiogenesis. CXCL12 is expressed at high levels in cancerous tissues; the main producer of CXCL12 is tumor-associated fibroblasts [7].

CXCR4, also known as CD184, is a functional receptor of CXCL12 and consists of 352 amino acids with seven membrane-splicing structures; this is an evolutionarily highly conserved G protein-coupled receptor (GPCR) [8]. Unlike other chemokine receptors, CXCR4 regulates inflammatory and immune processes by primarily acting on leukocytes [2]. CXCR4 is expressed in a variety of cell types, including lymphocytes, hematopoietic stem cells, endothelial cells, epithelial cells, stromal fibroblasts, and cancer cells; the expression of CXCR4 is upregulated under conditions of hypoxia, stress, and injury [9,10,11].

The binding of CXCL12 to CXCR4 can release free heterotrimeric G proteins into the cytoplasm that are composed of Gα, Gβ, and Gγ subunits. When the signaling pathway is activated, guanosine diphosphate (GDP) on the trimer is replaced by guanosine triphosphate (GTP); subsequently, the G protein is dissociated into a βγ dimer and an α monomer binding to GTP [12,13]. In turn, these activate various intracellular signal transduction pathways and downstream effectors that can trigger a range of biological phenomena, including cell survival, proliferation, migration and apoptosis, angiogenesis, actin polymerization, cytoskeletal rearrangement, and extracellular matrix remodeling [14,15,16,17,18].

### 2.2. Downstream Signaling Pathways of the CXCL12/CXCR4 Axis

**Figure 1:**
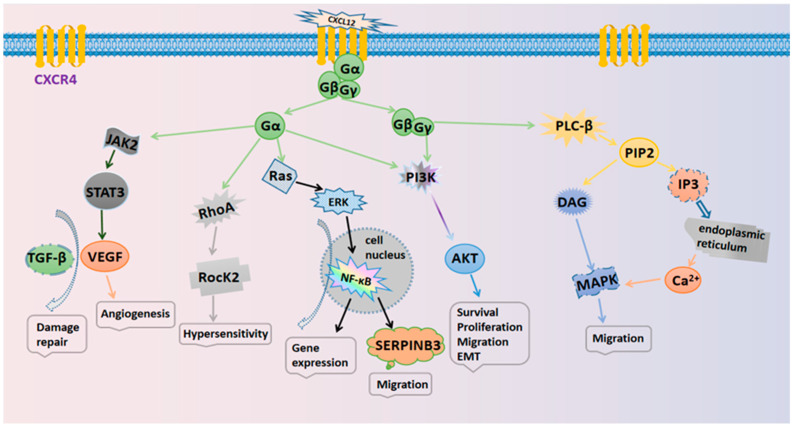
The signaling pathways in the CXCL12/CXCR4 biological axis.

The specific mechanisms responsible for the actions of the CXCL12/CXCR4 axis are illustrated in Figure 1. Several typical signaling pathways are involved. (i) Activation of the PI3K/AKT pathway mediates cell survival. The GβGγ dimer and Gα monomer can activate phosphoinositol-3 kinase (PI3K), thus leading to the phosphorylation of various signaling factors that can induce cell growth and survival by activating serine/threonine protein kinase AKT [2]. In cancer, the PI3K/AKT pathway plays an important role in promoting the epithelial–mesenchymal transition (EMT) and cell metastasis. (ii) Activation of the PLC/IP3 pathway mediates the release of the Ca^2+^ ion. The Gβγ subunit activates phosphatidylinositol-specific phospholipase C-β (PLC-β), which breaks down phosphatidylinositol 4,5-bisphosphate (PIP2) into inositol 1,4,5-trisphosphate (IP3) and diacylglycerol (DAG). IP3 can then bind to endoplasmic reticulum-specific receptors and promote the release of Ca^2+^ [19]. DAG and Ca^2+^ co-activate mitogen-activated protein kinase (MAPK), thus promoting cell migration [20]. (iii) Activation of the Ras/ERK pathway mediates gene expression. The Gα monomer activates extracellular signal-regulated kinase (ERK) through the Ras pathway; ERK then enters the nucleus and works with other regulatory proteins to activate cellular transcription factors that synergize with NF-κB to promote gene expression and cell cycle progression [21].

### 2.3. Mediated Physiological Effects in Tumors

The CXCL12/CXCR4 biological axis plays a crucial role in developmental processes in the body. During the early stages of development, CXCL12/CXCR4 signaling is involved in the recruitment of uterine natural killer cells, the migration of progenitor cells, placental formation, embryogenesis, and the development of the central nervous system and cardiovascular organogenesis [22]. In adulthood, CXCL12/CXCR4 signaling can influence the migration of stem cells in the bone marrow or ecotone to repair tissue damage [23]. In addition, the CXCL12/CXCR4 axis mediates a variety of physiological roles, including cell invasion, hematopoiesis, tissue repair, embryonic development, and immune cell trafficking [24,25]. The main physiological effects of CXCL12/CXCR4 signaling are given below.

1. *The regulation of cell growth and proliferation*. The CXCL12/CXCR4 axis can induce the proliferation of tumor cells via activation of the ERK and AKT signaling pathways. Tumor cells can express CXCL12 in a paracrine mode; this stimulates tumor stromal cells to produce tumor necrosis factors, thus promoting the growth of tumor cells [26].
2. *The regulation of the cell motility and migration*. The upregulation of CXCR4 on the cell surface allows the cells to efficiently recruit sources of chemokines. For example, CXCL12 can be expressed in the bone marrow (BM) and promote the migration of myeloma cells [27]. CXCL12/CXCR4 also enhances cell migration to promote the progression of human ovarian cancer [28].
3. *The mediation of cell adhesion*. Cell adhesion plays an important role in cell survival, migration, inflammation, angiogenesis, and injury repair. The CXCL12/CXCR4 axis can upregulate the expression of adhesion molecules such as very late antigen-4 (VLA-4) and very late antigen-5 (VLA-5), thereby increasing cell adhesion. VLA-4 increases the adhesion to fibronectin, thus resulting in an increase in overall cell adhesion. In addition, CXCL12 can promote the expression of adhesion molecules [29].
4. *Participation in angiogenesis*. Angiogenesis plays a critical role in normal development and the progression of cancer and is closely related to the CXCL12/CXCR4 axis. Activation of the CXCL12/CXCR4 axis prevents the degradation of β-catenin in the cytoplasm and the accumulation of β-catenin in the nucleus, which can induce angiogenesis via the Wnt/β-catenin, MAPK/ERK, and PI3K/AKT pathways [30]. The CXCL12/CXCR4 axis can also induce vascular endothelial growth factor (VEGF) expression through the JAK2/STAT3 pathway, inducing tumor angiogenesis [26].
5. *Mediating the repair of tissue damage*. The CXCL12/CXCR4 axis is crucial for stem cell homing and can recruit stem cells to the site of injury to then differentiate into functional cells to repair tissue damage. In addition, the CXCL12/CXCR4 axis can upregulate the expression of VEGF to mediate the repair of injured tissues together with transforming growth factor (TGF-β) [23].

### 2.4. The CXCL12/CXCR4 Axis and Cancer

In cancer, the CXCL12/CXCR4 biological axis can stimulate the proliferation and metastasis of tumor cells, regulate the inflammatory state of tumors, and activate the immune response within tumors by both autocrine and paracrine factors [31]. CXCR4 is expressed at high levels in many solid tumors and hematological tumors and represents an effective target for the diagnosis and treatment of tumors. In the next section, we focus on the role of the CXCL12/CXCR4 axis in the tumor microenvironment (TME) [32].

#### 2.4.1. Tumor Inflammation

Inflammation is one of the most important features of tumors [33]. Chronic inflammation can initiate and contribute to the development of tumors. In tumor tissue, certain inflammatory factors, such as mononuclear inflammatory cells (MICs) and myeloid-derived suppressor cells (MDSCs), can cause immunosuppression and thus promote tumor development [34]. Meanwhile, neutrophils, as the predominant leukocytes, often show contradictory therapeutic effects on cancers. Neutrophils could be polarized into different tumor-associated neutrophils (TAN) in the TME, for which N1-type TAN possesses the antitumor effects while N2-type TAN displays the pro-tumor effects [35,36].

Inflammation may promote tumor development via the CXCR4 pathway. In the TME, the inflammatory factor tumor necrosis factor α (TNF-α) activates NF-κB, which then enters the nucleus to upregulate the expression of CXCR4, thus promoting metastasis in human neuroblastoma [37]. On the surface of human tongue squamous cell carcinoma (TSCC) cells, the interaction of the pro-inflammatory cytokine IL-1β with IL-1 receptor 1 induces the activation of ERK and Notch signaling, both of which promote the expression of CXCR4 receptors, thus promoting tumor growth and metastasis [38]. In another study, Zhang et al. reported that miR-302b, a gene that can inhibit CXCR4, efficiently downregulated cancer-related inflammation (CRI) in patients with esophageal cancer (EC) [39].

#### 2.4.2. Tumor Immunity

The CXCL12/CXCR4 axis plays a key role in innate and adaptive immune responses, mediates the retention of hematopoietic stem cells in the bone marrow, and is responsible for the transport of T-cell precursors to the thymus and the clearance of neutrophils from the bone marrow. The CXCL12/CXCR4 axis mediates the metastasis and homing of immune cells at tumor sites, thus affecting the specific immune response. In the TME, high expression levels of CXCR4 increase the infiltration of myeloid-derived suppressor cells (MDSCs) but reduces CD8+ T cell infiltration, thus inhibiting the immune response [40]. CXCR4 also increases Treg infiltration in tumors and releases an immunosuppressor of T cells by suppressing IFN-γ production, promoting proliferation, and inhibiting apoptosis [41].

In colorectal cancer cells, the CXCL12/CXCR4 axis regulates PTEN to induce M2 polarization by activating the PI3K/Akt signaling pathway. M2-polarized macrophages promote cancer metastasis by promoting EMT and the secretion of VEGF [42]. Using an animal model of ovarian cancer, Righi et al. found that the CXCR4 antagonist AMD3100 reduced the infiltration of regulatory T cells (Treg) and significantly enhanced the T cell-mediated anti-tumor immune response [43]. In another animal-based study, Yang et al. reported that CXCR4 inhibition on myeloid cells upregulated the secretion of cytokine IL18 and inhibited the Fas/FasL signaling pathway; this promoted the neutrophil-dependent activation of NK cells, thus enhancing anti-tumor immunity and inhibiting tumor growth [44].

#### 2.4.3. Tumor Cells

The CXCL12/CXCR4 axis can interact with multiple cellular signaling pathways via both local autocrine and paracrine mechanisms. In this context, the CXCL12/CXCR4 axis plays a decisive role in the development and progression of cancer, including cell proliferation and metastasis, tumor angiogenesis, the epithelial mesenchymal transition, intracellular calcium increase, and gene transcription [45,46,47].

CXCR4 signaling can prolong the lifespan of stem cells and increase the potential for DNA damage and mutations, thus transforming these cells into cancer stem cells (CSCs), ultimately promoting the progression of cancer [23]. The breast tumor leader cells (K14+) can utilize both chemical and mechanical cues by CXCR4 signaling, including low oxygen, collagen density, chemokine gradient, and interstitial fluid flow, in order to guide collective migration [48]. In gastric cancer cells, CXCL12/CXCR4 signaling can activate NF-kB; this upregulates the expression of serine protease inhibitor branch B member 3 (SERPINB3), thus promoting the metastasis of tumor cells [49]. In bone cancer, CXCR4 mediates pain hypersensitivity by activating the downstream RhoA/ROCK2 pathway [50]. In pancreatic ductal adenocarcinoma (PDAC), the CXCL12/CXCR4 axis mediates the desmoplastic reaction; this changes the tumor mechanical microenvironment and promotes drug resistance [51]. In addition, the CXCL12/CXCR4 biological axis plays an important role in the progression of several cancers, including ovarian cancer and squamous cell carcinoma [52,53]. The CXCR4 antagonist GST-NT21MP has been shown to block CXCR4 receptors and inhibit Src activation, thus inhibiting downstream Akt, FAK, and ERK signaling; these events inhibit tumor growth and the metastasis of breast cancer [54].

## 3. CXCL12/CXCR4-Based Antagonists

When considering the significant role of the CXCL12/CXCR4 axis in cell growth, proliferation, and migration, it is clear that the inhibition or blockade of CXCL12/CXCR4 holds promising therapeutic prospects for several diseases but especially for cancer therapy. CXCR4 antagonists can block CXCL12-dependent growth and proliferation signals, inhibit tumor cell metastasis and angiogenesis, and reduce drug resistance in tumor cells [55]. Therefore, the discovery and synthesis of antagonists that exert action on the CXCL12/CXCR4 axis have become a significant focus of research. CXCL12/CXCR4-based antagonists are available in several different forms, including small-molecule compounds, peptides, antibodies, microRNAs, and natural products; more than 10 of these have entered clinical trials (see Table 1).

### 3.1. Small-Molecule Compounds

**Figure 2:**
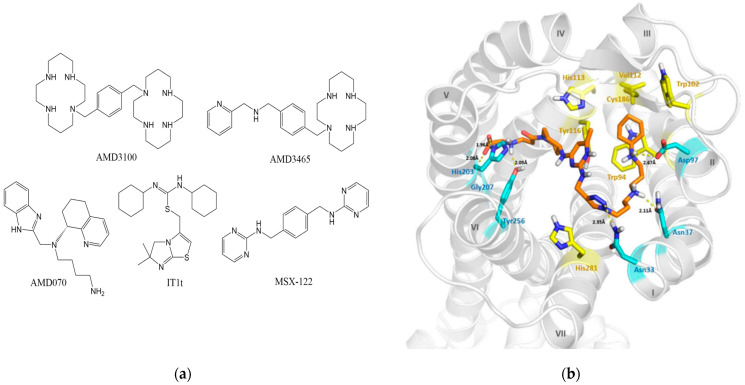
(**a**) The representative chemical structures of small-molecule compounds CXCL12/CXCR4 antagonists; (**b**) the X-ray structure of CXCR4 in interaction with its antagonist (I–VII belong to the different fragments of CXCR4 protein).

Small-molecule compounds that exert action as CXCL12/CXCR4-based antagonists have significant potential for development and commercialization. Thus far, dozens of small-molecule compounds have been studied and entered clinical trials. The chemical structures of some representative small-molecule compounds are given in Figure 2a. We also show the X-ray structure of CXCR4 interacting with one of its antagonists in Figure 2b [75].

#### 3.1.1. Plerixafor

Plerixafor, also known as AMD3100, is a small non-peptide molecule that specifically antagonizes the CXCR4 receptor. The primary structure of AMD3100 contains two cycloglycan rings linked by a phenylene linker. Under physiological conditions, the protonated nitrogen atom on the ring interacts with the carboxylic acid group on CXCR4. This inhibits the binding of CXCL12 to CXCR4, blocks downstream signaling, and regulates various physiological activities [13]. AMD3100 is the only marketed CXCL12/CXCR4 antagonist and was approved by the FDA in 2008 for autologous transplantation in patients with non-Hodgkin’s lymphoma (NHL) and multiple myeloma (MM).

The therapeutic effects of AMD3100 have been demonstrated in a variety of cancers. AMD3100 can block the activation of protein kinase Akt and extracellular signal-regulated kinase 1/2 (ERK1/2), both of which inhibit the survival, proliferation, and migration of tumor cells [76]. Moreover, immunosuppression can also be eliminated by suppressing PD-1 expression on CD8+ T cells and Treg cell transduction within the tumor [77]. Using an animal model, Liao et al. found that AMD3100 could reduce the survival and metastasis of osteosarcoma by inhibiting the c-Jun NH 2-terminal kinase (JNK) and AKT pathways [78]. Using another animal model, Kioi et al. found that AMD3100 inhibited angiogenesis and tumor recurrence by hindering the recruitment of bone marrow-derived dendritic cells (BMDC) in glioblastoma [79]. AMD3100 also enhanced the radiosensitivity of triple-negative breast cancer (TNBC) cells, thus improving the efficacy of radiotherapy for TNBC [80].

The combination of AMD3100 with other drugs has also been investigated with good efficacies. For example, when combined with the steroid hormone mifepristone, AMD3100 inhibited actin polymerization by downregulating the expression of MMP-2, MMP-9, cyclooxygenase-2, and vascular growth factor, thus significantly reducing cell proliferation and migration [81]. In the treatment of prostate cancer, the combination of resveratrol and AMD3100 impeded the EMT of cancer cells and increased the expression of apoptosis-related genes, herein inhibiting the metastasis of cancer cells [82]. The combination of colony-stimulating factor (G-CSF) and AMD3100 mobilized bone marrow-derived stem cells (BMSCs), thus preventing cisplatin-induced acute tubular injury, thereby improving renal function [83].

#### 3.1.2. Analogs of AMD3100

Different analogs of AMD3100 have been synthesized and evaluated over the last few decades. The first cycloglycan ring of AMD3100 can be replaced by pyridine methylene to obtain an analog known as AMD3465. AMD3465 has the same biological properties as AMD3100, blocks the binding of CXCL12 to CXCR4, and inhibits CXCL12-mediated intracellular calcium signaling, CXCR4 endocytosis, and the activation of MAPK signaling; furthermore, the antagonistic activity of AMD3465 is ten-fold higher than that of AMD3100 [84]. Using an animal model, Ling et al. showed that AMD3465 acted on tumor cells and immune cells by regulating the STAT3 signaling pathway, thereby inhibiting breast cancer growth and metastasis [85].

Mavorixafor, also known as AMD070, is also a CXCR4 metamorphic inhibitor with good tolerability and oral bioavailability; this can effectively inhibit the replication of X4 HIV-1. Using an animal model, Uchida showed that AMD070 significantly inhibited CXCL12/CXCR4-dependent migration and the invasion of oral cancer cells [86]. In addition, nanobubbles created by the combination of AMD070 with the light-absorbing material indocyanine green (ICG) were found to block the interaction of CXCL12 with CXCR4 in breast cancer cells, thereby inhibiting the growth of cancer cells and promoting the apoptosis [87].

#### 3.1.3. IT1t

IT1t is a small isothiourea derivative-like molecule that selectively interferes with CXCR4 dimers and oligomers [88]. Animal studies performed by Tulotta et al. found that IT1t could affect the early metastasis of TNBC [89]. In addition, IT1t was the only small molecule that could co-crystallize with CXCR4 and AMD3100. IT1t was also shown to inhibit TLR7-mediated type I interferon (IFN) signaling in human plasmacytoid dendritic cells (PDCs) from the blood and tonsils, thereby reducing inflammation in patients with systemic lupus erythematosus (SLE) [90].

#### 3.1.4. MSX-122

The small molecule MSX-122 is a partial inhibitor that can block the functions of CXCR4 by competitively binding to the CXCR4 receptor. MSX-122 blocks cAMP signaling, inhibits cell recruitment and homing without mobilizing stem cells, and also exerts anti-inflammatory and anti-tumor metastatic effects [91]. MSX-122 can block lung metastases from breast cancer and squamous cell carcinoma of head and neck (SCCHN), block liver metastases from uveal melanoma, and attenuate radiation-induced pulmonary fibrosis (PF) [92].

### 3.2. Peptides

#### 3.2.1. T140

T140, a synthetic small-molecule peptide, is a CXCR4 inverse agonist that reduces autonomous CXCR4 signaling in constitutive CXCR4 mutants and has high CXCR4 affinity and anti-HIV activity [93]. T140, and its analogs, inhibit actin polymerization and chemotaxis, suppress CXCL12-induced MAPK activation and STAT3 serine phosphorylation, and impede the migration of chronic lymphocytic leukemia (CLL) cells to stromal cells [94].

T140 is often modified to improve its stability in serum. C-terminal amidation and citrulline substitution in T140 can produce TN14003 ([Cit6]-T140 and C-terminal amide) and TC14012 ([Cit6, D-Cit8]-T140 and C-terminal amide), respectively; both of these have high affinity for CXCR4 and are stable in the serum. TN14003, also known as motixafortide, is a potent CXCR4 inhibitor that reduces the migration and invasion of pancreatic cancer cells by impeding CXCL12-dependent MAPK activation [95]. Furthermore, TN14003 can reduce the secretion of MMP-3, MMP-9, and MMP-13 in cartilage tissue, aggregate proteoglycans, and reduce the degradation of collagen II, thus relieving degeneration of the articular cartilage [96]. BKT140, an F-benzoylated isomer of TN14003, is also a CXCR4 antagonist that induces the mobilization of hematopoietic stem and progenitor cells in a dose-dependent manner [62]. BKT140 reduces the growth of acute myeloid leukemia and multiple myeloma [97], disrupts the CXCL12-mediated adhesion of tumor cells to stromal cells to improve the efficacy of radiotherapy and chemotherapy in lung cancer [98], and plays a prominent role in the treatment of lymphoma.

#### 3.2.2. CTCE-9908

CTCE-9908 is a CXCL12 analog composed of 17 amino acids that can bind competitively to CXCR4. CTCE-9908 inhibits tumor cell invasion and tumor angiogenesis via the CXCL12/CXCR4 axis [99]. In addition, CTCE-9908 inhibits AKT activity, and when combined with docetaxel, can effectively inhibit primary tumor growth. In addition, the combination of CTCE-9908 and the anti-angiogenic drug DC101 was shown to target angiogenesis and inhibit tumor metastasis [100]. In vitro research by Kwong et al. showed that CTCE-9908 mediated the multinucleation of ovarian cancer cells and blocked the G2-M phase in the cell cycle by attenuating DNA damage checkpoints and spindle assembly checkpoints and inducing abnormal mitosis; these events impeded the growth and migration of ovarian cancer cells [101]. In addition, CTCE-9908 has been shown to affect the growth and metastasis of osteosarcoma and melanoma [102].

#### 3.2.3. LY2510924

LY2510924, a small cyclic peptide containing nonnatural amino acids, is a potent and selective CXCR4 antagonist with good stability in vivo. LY2510924 attenuates the proliferation and chemotaxis of leukemic cells by inhibiting CXCL12-mediated PI3K/AKT and MAPK pro-survival signaling; it can also reverse matrix-mediated drug resistance [103]. LY2510924 inhibits the lung metastasis of breast cancer cells by blocking the migration and homing of tumor cells and plays a dose-dependent inhibitory role in various other solid tumor models [104]. The multi-functional scaffold generated by c-terminal modification of LY2510924 can be applied to CXCR4-targeted probe development, tumor imaging, and drug-targeted therapy [105].

#### 3.2.4. Balixafortide

Balixafortide, also known as POL6326, is a potent macrocyclic peptide of a CXCR4 antagonist that exhibits good safety and tolerability profiles and can achieve dose-dependent cell mobilization. POL5551, an analog of POL6326, is a synthetic protein epitope mimic (PEM) with efficient mobilization capacity at optimal doses [106]. POL5551 can mediate angiogenesis, tissue repair, and improve cardiac function after myocardial infarction via the action of splenic Foxp3 regulatory T (Treg) cells [107].

### 3.3. Antibodies

#### 3.3.1. Ulocuplumab

Ulocuplumab, also known as BMS-936564 or MDX-1338, is a humanized IgG4 monoclonal antibody. As a potent CXCR4 antagonist, ulocuplumab can effectively block the binding of CXCL12 to CXCR4, thus inhibiting F-actin polymerization, calcium flow, and cell migration [108]. Ulocuplumab lacks complement-dependent cytotoxic (CDC) and antibody-dependent cell-mediated cytotoxic (ADCC) activity; in contrast, ulocuplumab induces apoptosis by binding directly with CXCR4 [109].

#### 3.3.2. PF-06747143

PF-06747143, a novel humanized IgG1 CXCR4 antagonist antibody, selectively binds to the CXCR4 receptor and effectively inhibits the CXCL12/CXCR4 signaling pathway. Compared to IgG4 antibodies, PF-06747143 exerts the unique Fc effector functionality (ADCC and CDC) to kill tumor cells; this leads to improved efficacy [68]. Using an animal model, Kashyap et al. demonstrated that PF-06747143 played a role in the production of reactive oxygen species (ROS) through antibody bivalency, thereby mediating malignant CLL-B cell death without the activation of cysteine aspartate protease [110]. The combination of PF-06747143 with standard-of-care (SOC) drugs could lead to improved therapeutic effects when treating hematological tumors.

#### 3.3.3. LY2624587, hz515H7, and 30D8

LY2624587 is a humanized monoclonal antibody that effectively blocks the binding of CXCL12 to CXCR4, thus inhibiting the transduction of its downstream signaling pathways (ERK and AKT pathways). LY2624587 has no agonist activity, but does have clinical potential as a single agent or in combination with other drugs for the treatment of hematological tumors [111]. Hz515H7, a humanized IgG1 monoclonal antibody, competitively binds to CXCR4, thereby inhibiting G protein activation and β-arrestin-2 recruitment. Hz515H7 can trigger ADCC and CDC effector functions to inhibit the proliferation and migration of cancer cells [112]. 30D8 is also a high-affinity CXCL12 antibody that can prevent binding to CXCR4 by competitively binding to CXCL12α; 30D8 can also inhibit primary tumors alone or in combination with VEGF antibodies [113].

### 3.4. Natural Products

It has been verified that many natural products can produce anti-cancer and anti-viral effects through the CXCL12/CXCR4 axis, including flavonoids, isoflavones, bioketones, and isoprenoidyl flavonoids [114]. For example, Rosa et al. identified parazoanthines, a hydantoin alkaloid that was extracted from anemones in the Mediterranean Sea, as a novel CXCR4 antagonist, by combining experimental and computational approaches [115]. Luteolin (L), ellagic acid (E), and punic acid (P), all isolated from pomegranate juice (PJ), have been shown to act on the CXCL12/CXCR4/AKT signaling axis to stimulate cell adhesion, thus inhibiting the metastasis of prostate cancer cells [116]. Penicillixanthone A (PXA), a marine-derived natural flavonoid dimer, is a dual CCR5/CXCR4 co-receptor antagonist with good anti-HIV-1 activity [117]. The pentacyclic triterpene polyformic acid (PA), obtained from *Euscaphis japonica*, inhibits the transcription and expression of the CXCR4 protein in breast cancer cells and suppresses CXCL12-induced cell invasion [118]. The triterpenoid glycoside sakosaponin A (SSA) in *Radix bupleuri* has been shown to inhibit CXCR4 protein expression and thus suppress the migration and invasion of triple-negative breast cancer cells [70]. Naringin, the main active ingredient in the Chinese herb *Drynaria fortunei*, promotes the proliferation of endothelial cells and neoangiogenesis via the CXCL12/CXCR4/PI3K/Akt signaling pathway [71]. Generally, the study of natural products acting on the CXCL12/CXCR4 axis is becoming increasingly widespread and is becoming an important area of research.

### 3.5. microRNAs

MicroRNAs (miRNAs) are a class of non-coding single-stranded RNA molecules that are approximately 22 nucleotides in length. These are encoded by endogenous genes and are involved in the post-transcriptional regulation of gene expression. Certain microRNAs have been bio-engineered and play a crucial role in the treatment of tumors by acting on the CXCL12/CXCR4 axis. For example, miR-146 inhibits CXCR4 expression, thus hindering the proliferation and migration of colorectal cancer cells [72,73]. miR-193-5p is known to impede the proliferation of colorectal cancer cells by inhibiting the expression of CXCR4 [74].

## 4. The Action of Nano-Based Drug Delivery Systems on the CXCL12/CXCR4 Axis

Nano-based drug delivery systems (NDDS) have attracted significant concerns with regard to their potential application in cancer therapy over the last few decades [119]. For conventional chemotherapeutic drugs, the most significant drawbacks of NDDS relate to unsatisfactory therapeutic effects and serious adverse drug reactions; these are closely associated with poor stability, multi-drug resistance, and uncontrolled systemic distribution [120]. Therefore, research in the field of NDDS is being developed rapidly. These nano-formulations possess many advantages such as optimized pharmacokinetic profiles, controlled drug release, improved accumulation at the tumor site, enhanced anti-tumor efficacy, and a reduced risk of adverse reactions [121]. In addition, the beneficial mechanical properties of nanocarriers can improve the efficiency of drugs to target tumors [122]. At the present time, numerous products have been approved into market, including doxorubicin hydrochloride liposomes injection (Doxil), liposomal amphotericin B dry powder, and paclitaxel liposomes for injection. Of these, the action of NDDS on the CXCL12/CXCR4 axis has been tested extensively; some representative nano-formulations are summarized in Table 2.

### 4.1. Liposomes

Liposomes are microvesicles that consist of lipid bilayers with particle sizes generally ranging from 20 to 1000 nm; those that are <200 nm are particularly suitable for intravenous administration. Liposomal systems can improve drug solubility, control drug release, and provide drug targeting options [141]. Because of their structural similarity to cell membranes, liposomes possess desirable physiological environmental stability and biocompatibility [142]. In addition, liposomes can be further functionalized with surface ligand modifications to realize active targeting ability [143].

Li et al. prepared liposomes encapsulated with IR780 by solvent evaporation and then co-incubated these liposomes with AMD3100; this led to AMD3100 being coated on the surface of the liposomes by electrostatic adsorption, thus producing IR780-AMD-NLCs [124]. In a mouse model of breast cancer, the AMD3100-coated liposomes coating led to an improvement in tumor targeting in vivo and accumulation at the tumor site; these events completely inhibited CXCR4 receptors, thus hindering the invasion and lung metastasis of breast cancer cells. This nano-formulation, featuring IR780 liposomes coated with AMD3100, effectively mediated active targeting and photothermal therapeutic effects.

Caterina et al. prepared DOX-loaded liposomes by a thin film dispersion method. Then, peptide R (CXCR4 antagonistic cyclic peptide) was incubated with the DOX-loaded liposomes overnight to obtain generate R-modified stealth liposomes (PL-Peptide R). The PL-Peptide R efficiently inhibited CXCR4-dependent cell migration and lung metastasis in a mouse model [125]. Compared to free Peptide R, liposomes led to increased stability in vivo and inhibited CXCR4 more efficiently by generating multi-valent binding ligands, thus inhibiting the metastasis of cancer cells.

### 4.2. Nano Micelles

Polymeric micelles are nanostructures that are produced by the self-assembly of amphiphilic copolymers; it is possible to load insoluble drugs into the inner hydrophobic core in a very efficient manner. Optimal loading of drugs and improved stability can be achieved by varying the combination of copolymers [144]. Polymeric micelles have a wide range of applications in gene delivery and diagnostic imaging, largely due to their small size, good permeability, and retention effects in living organisms [145].

Meng et al. co-dissolved E5 (chemically synthesized CXCR4 antagonist) powder and DSPE-PEG2000 in 5% glucose solution, which were then self-assembled by sonication and vortexing to generate nanomicelles (M-E5) [126]. In a mouse model of human acute myeloid leukemia (AML), M-E5 exhibited good physiological stability and was able to target and block CXCR4 on the surface of leukemic cells to inhibit mediated cell migration. Accordingly, M-E5 effectively inhibited cell proliferation and promoted apoptosis via the CXCL12/CXCR4 signaling pathway to play a therapeutic role in AML. In addition, M-E5-Dox micelles were further prepared and co-loaded with E5 and Dox; these structures could efficiently inhibit the migration of MCF-7 and HepG2 tumor cells by blocking the phosphorylation of Akt, Erk, and MAPK proteins [127].

### 4.3. Nanoparticles

Nanoparticles are solid colloids with particle sizes ranging from 10 to 1000 nm and are prepared by non-toxic and biodegradable nanocarriers. Nanoparticles possess the advantages of a high drug loading capacity and good stability, and have a wide range of potential applications in the field of tumor therapy [146]. The cellular uptake of nanoparticles can be improved by regulating the surface charge and modifying the targeting recognition group.

Hanh et al. prepared nanoparticles loaded with miR-200c (PD-L1 immune checkpoint inhibitor) using PCL-PEI as a nanocarrier. Afterward, PGA-pep was synthesized by the conjugation of PGA and LY2510924 peptide (CXCR4 antagonist) and then cross-linked with the nanoparticles to obtain novel miR@PCL-PEI/Dab@PGA-pep nanoparticles [128]. In a mouse model of colon carcinoma, compared to nontargeted nanoparticles, nanoparticles that had been modified with CXCR4 antagonists had a higher affinity for cancer cells, an improved cellular uptake and cytotoxicity of the nanoparticles, and showed a greater aggregation at tumor sites; these events activated the immune response against the tumor.

Anissa et al. prepared poly (lactic acid-co-glycolic acid) (PLGA)/Pluronic F127 nanoparticles by microjet-assisted nanoprecipitation [129]. These were further modified with biotinylated CXCL12 to obtain CXCL12-PLGA/Pluronic NPs. CXCL12-NPs specifically recognized CXCR4+ THP-1 monocytic leukemia cells and were internalized effectively; however, the nanoparticles did not induce cell chemotaxis and cell migration was significantly hindered.

In another study, Xue et al. generated AMD-PEG-NHS by the polymerization of Mal-PEG-NHS with AMD3100-8HCl and an addition reaction; this was then linked to BSA via an amide bond to obtain AMD-PEG-BSA nanoparticles [130]. In addition, PTX was encapsulated in nanoparticles by a biomineralization method to obtain the final nano-formulation: AMD-PEG-BSA-NP-PTX. In a mouse model of ovarian cancer, AMD-PEG-BSA-NP-PTX exhibited a good safety profile and in vivo biocompatibility. The nanoparticles could target Caov3 cells after modification with AMD3100 and were effectively internalized and aggregated efficiently at the tumor site. Furthermore, these nanoparticles inhibited cancer cell proliferation and metastasis by regulating the EMT process and the NF-κB pathway, thus providing a new pathway for the treatment of breast cancer.

### 4.4. Nanocomplexes

Nanocomplexes are composite drug delivery systems with a size at the nano-level. These can disperse drugs uniformly in a carrier material by appropriate preparation methods. Nanocomplexes have a large specific surface area with high drug-carrying capacity, and they can increase drug solubility and improve drug stability and bioavailability. In addition, by modulating their surface properties, nanocomplexes can achieve efficient cellular uptake. Thus, nanocomplexes can serve as efficient nanocarriers of bioactive molecules and play a crucial role in the sustained and controlled release of drugs [147].

Xue et al. synthesized CM-COOH using CM (CXCR4 antagonist) and succinic anhydride, thus generating Dex-PEI (DP) using Dex-COOH and PEI. Afterward, DPC was obtained by the conjugation between DP and CM-COOH; this was further co-incubated with miR-34a to obtain DPC/miRNA nanocomplexes by electrostatic adsorption [131]. Due to the specific targeting effect of CM, these nanocomplexes improved the efficacy of miRNA delivery in a mouse model of breast cancer by improving cellular uptake. Furthermore, these nanocomplexes significantly inhibited CXCR4 and down-regulated CD44 expression, thus exhibiting a potent tumor-killing ability and inhibition of the metastatic and invasive activity of cancer cells. This proposed nano-formulation provides a new approach for the treatment of metastatic tumors.

Wang et al. synthesized PAMD from AMD3100 and N,’-Hexamethylenebisacrylamide (HMBA) by Michael addition to generate PAMD-Ch. Differing degrees of cholesterol substitution were achieved by amide bonding with cholesteryl chloroformate under certain conditions. Next, PAMD-Ch/siRNA nanocomplexes were prepared by the co-incubation of PAMD-Ch and siRNA. In a human cellular model of epithelial osteosarcoma, AMD-3100-derived PAMD exhibited significant cationic properties and was a favorable vector for siRNA that could promote the efficient transfection of siRNA in cancer cells. Furthermore, AMD3100 maintained its significant CXCR4 inhibitory effect, thus impeding metastasis and the invasion of cancer cells [132].

Aïda et al. covalently bound a nanocarrier containing T22 to maleimide functionalized monomethyl auristain E (MC-MMAE) via alkylamine bonds to generate T22-AUR nanocomplexes in which T22 selectively targeted the CXCR4 receptor [133]. T22-AUR exhibited mean particle sizes of 17.9 ± 0.7 nm and contained approximately 12 CXCR4 ligands per nanoparticle; these particles efficiently targeted CXCR4 receptors and were taken up efficiently by cells; these particles exhibited significant cytotoxic effects by inducing G2/M mitotic block, DNA damage, and mitotic mutations. In a mouse model of diffuse large B-cell lymphoma (DLBCL), this nanocomplex effectively blocked the spread and metastasis without obvious off-target toxicity.

### 4.5. Inorganic Nanoparticles

In recent years, inorganic nano-drug delivery systems have been rapidly developed due to their unique structure and properties. Typical inorganic nanomaterials include silica nanoparticles, gold nanoparticles, magnetic nanoparticles, quantum dots, and carbon nanotubes [148]; these are all widely used for the delivery of anti-cancer drugs, the construction of targeted drugs, and the diagnostic imaging of diseases. In addition, inorganic–organic hybrid nano-systems provide new concepts and pathways for the fields of drug delivery, photodynamic therapy, bio-detection, and vaccine development [149].

One form of novel inorganic hybrid nanoparticles was loaded with CXCR4 siRNA molecules and prepared by Khaled et al. using a one-pot synthesis approach [134]. This nano-formulation was generated by the free radical polymerization reaction of a nanostructured inorganic silica core with an organic pH-responsive hydrogel shell poly (2-diethylaminoethyl methacrylate) (PDEAEM). In a mouse model of human breast cancer, these nanoparticles improved the biostability of siRNA and preferentially accumulated at the tumor site due to pH-responsive properties, thus delivering siRNA to the cytoplasm and acting directly on CXCR4 transcripts, thereby significantly inhibiting CXCR4 protein expression in breast cancer cells.

Zhao et al. prepared an AMD3100 (Plerixafor) functionalized and targeted gold nanocluster (64CuAuNCs-AMD3100) [135]. AMD3100 acted as a CXCR4 antagonist with high affinity to CXCR4 receptors. In a mouse model of breast cancer, when compared with ligand tracers alone (64Cu-AMD3100) and non-targeted nanoclusters (64CuAuNCs), 64CuAuNCs-AMD3100 exhibited higher levels of sensitivity and accuracy, thus demonstrating efficient targeting ability to CXCR4 receptors when highly expressed in early-stage tumors as well as metastases, thus providing a new concept for the early diagnosis of tumors.

### 4.6. Biomimetic Nanoparticles

Biomimetic nanoparticles are a new form of drug delivery system obtained by the special integration of bionic materials (such as cell membranes, proteins, bacteria, and viruses) with organic or inorganic nanocarriers [150,151]. This form of nanoparticles could improve histocompatibility, reduce immunogenicity, and provide drug targeting by mimicking the structure and function of a specific organism [152]. For example, cell membranes (of red blood cells, tumor cells, and immune cells) are widely used to modify nanoparticles to avoid their clearance by immune cells and to prolong their circulation time and improve targeting efficiency [153].

Luo et al. reacted p-hydroxybenzyl alcohol (HBA), oxalyl chloride (OC), and poly-(ethylene glycol) 2000 (PEG2000) by polycondensation polymerization to obtain the ROS-responsive amphiphilic copolymer HBA-OC-PEG2000 (HOP). Rapamycin (RAPA) was first encapsulated into HOP NPs by a dialysis method. Then, the NPs were wrapped using CXCR4-overexpressing primary mouse thoracic aorta endothelial cell (PMTAEC) membranes to obtain RAPA@BMHOP [136]. In a mouse model of middle cerebral artery occlusion (MCAO), when compared to nontargeted nanoparticles, RAPA@BMHOP exhibited a three-fold increase in cell delivery efficiency, extended the in vivo circulation time, and increased stability.

MC-3T3 cells with high expression levels of the CXCR4 membrane receptor were obtained by transfection with a CXCR4 recombinant lentivirus [137]. This was followed by vortexing and gradient centrifugation to extract cell membrane vesicles (CXCR4-CMVs). Wang et al. then prepared biomimetic nanoparticles (CXCR4/Cur-CMVs) by loading curcumin into the CXCR4-CMVs by physical entrapment. In mouse models of inflammatory disease, these nanoparticles significantly improved homing and exerted an impact on inflammatory tissues by targeting the CXCR4/CXCL12 axis, thus demonstrating improved targeting and anti-inflammatory effects.

Bose et al. designed bioengineered stem cell membrane-functionalized nanocarriers (BSMNCs) [138]. VEGF-poly (lactic-1 co-glycolic acid) (PLGA) nanocarriers (PNCs) were first prepared using a double-emulsion solvent-evaporation method. These were then mixed with human adipose-derived stem cell membranes that overexpressed CXCR4 (CXCR4-hASCs) to obtain BSMNCs by sonication. In a mouse model of hindlimb ischemia, when compared with uncoated PNCs, BSMNCs effectively reduced the immunological clearance by immune cells and significantly improved the targeting ability and blood perfusion to the ischemic site.

### 4.7. Other Forms of Nano-Carriers

In addition to the above typical nano-formulations, there are other nano-carriers that have been extensively studied, such as nano-gels and nano-emulsions [154].

Zhang et al. prepared a dextrin nanogel, DOX-AMD-DNG, which was loaded with DOX and coated by AMD3100 using a suspension method [139]. DOX-AMD-DNG could target the CXCR4 receptor and inhibit tumor metastasis. In a mouse model of breast cancer, when compared to DOX-DNG, DOX-AMD-DNG exhibited more efficient 4T1 breast cancer cell targeting, uptake efficiency, and higher cytotoxicity.

Li et al. conjugated heptafluorobutyric anhydride (HFBA) and AMD3100 by Michael addition, which was then fluorinated to obtain FM. Subsequently, siRNA was adsorbed to generate a nano-emulsion: FM@PFC/siRNA [140]. In a mouse model of breast cancer, this nano-emulsion inhibited CXCR4 and STAT3 expression with significant anti-metastatic activity. In addition, this system increased IL-12 expression and decreased Foxp3 expression during tumor metastasis and promoted immune activation in the metastatic microenvironment.

## 5. Outlook

The CXCL12/CXCR4 biological axis plays a unique role in a variety of diseases and cancers. The development and application of drugs based on this biological axis have been proven already by clinical studies. With the development of nanotechnology, nano-formulations of CXCR4 antagonists are being increasingly applied for the treatment of various cancers and aim to improve drug stability and bioavailability for better therapeutic effects. However, the specific regulatory mechanisms of the CXCL12/CXCR4 biological axis have yet to be fully elucidated and its antagonists still need to be investigated further.

## Figures and Tables

**Table 1:** Typical CXCR4 antagonists and their categories.

Categories	Drugs	Molecular Weight	Diseases	Routes of Administration	Status	NCTs	Refs
Small-molecule compound	Plerixafor	502.78	Autologous Stem Cell Transplant	Subcutaneous	Approved	NCT00291811	[56]
			Acute myeloid leukemia	Oral	Phase I/II	NCT00512252	[57]
			Lymphoma	Subcutaneous	Phase III	NCT00103610	[58]
Small-molecule compound	Mavorixafor	349.47	HIV infection	Oral	Phase I/II	NCT00089466	[59,60]
			Waldenstrom’s Macroglobulinemia	Oral	Phase I	NCT04274738	[61]
Peptide	Motixafortide	2159.52	Multiple myeloma	Subcutaneous	Phase I/II	NCT01010880	[62]
			Multiple myeloma	Subcutaneous	Phase III	NCT03246529	[63]
Peptide	Balixafortide	1864.11	Metastatic breast cancer	Intravenous	Phase I	NCT01837095	[64]
Peptide	LY2510924	1189.45	Small Cell Lung Carcinoma	Subcutaneous, Intravenous	Phase II	NCT01439568	[65]
			Metastatic Clear Cell Renal Cell Carcinoma	Subcutaneous	Phase II	NCT01391130	[66]
Antibody	Ulocuplumab	/	Waldenstrom’s Macroglobulinemia	Intravenous	Phase I/II	NCT03225716	[67,68]
Antibody	PF-06747143	/	Acute myelogenous leukemia	Intravenous	Phase I	NCT02954653	[69]
Natural product	Saikosaponin A	780.98	Triple-negative breast cancer	Intravenous	Pre-clinical	/	[70]
Natural product	Naringin	580.53	Ischemic diseases	Subcutaneous	Pre-clinical	/	[71]
MicroRNA	miR-146	/	Colorectal cancer	Self-expression	Pre-clinical	/	[72,73]
MicroRNA	miR-193-5p	/	Colorectal cancer	Self-expression	Pre-clinical	/	[74]

NCTs: National Clinical Trials.

**Table 2:** Typical nano-drug delivery systems.

Drug Delivery Systems	Nanocarriers	Drugs	Diseases	Refs
Liposomes	PC, Chol	AMD3100, Pirfenidone	Liver fibrosis	[123]
	PC, MCT	AMD3100, IR780	Breast cancer	[124]
	DPPC, Chol, DSPE-PEG2000	Peptide R, DOX	Solid tumors	[125]
Nano micelles	DSPE-PEG	Peptide E5, DOX	AML	[126,127]
Nanoparticles	PCL-PEI, (OPSS)-PEG-NHS	LY2510924, miR-200c, Dabrafenib	Colon cancer	[128]
	PLGA, Pluronic F127	Biotinylated CXCL12	Leukemia	[129]
	Mal-PEG-NHS, BSA	AMD3100, PTX	Ovarian cancer	[130]
Nanocomplexes	Dextrin-PEI-CM	CM, miR-34a	Metastatic breast cancer	[131]
	PEI, HMBA, DIPEA	AMD3100, siRNA	Osteosarcoma	[132]
	T22-GFP-H6, MC-MMAE	T22	Lymphoma	[133]
Inorganic Nanoparticles	PDEAEM, VTMS	siRNA	Breast cancer	[134]
	64CuAuNCs, TA-PEG	AMD3100	Breast cancer	[135]
Biomimetic Nanoparticles	HBA-OC-PEG2000, CMs	Rapamycin	Cerebral ischemia-reperfusion injury	[136]
	CMVs	Curcumin	inflammation	[137]
	VEGF-(PLGA), CXCR4-hASCs	VEGF	Critical limb ischemia	[138]
Nano-gels	Dex-COOH, Dex-SH	AMD3100, DOX	Breast cancer	[139]
Nano-emulsions	HMBA, HFBA, PFC	AMD3100, siRNA	Lung Metastases	[140]

## Data Availability

Not applicable.
